# Quantitative analysis of the methane gas emissions from municipal solid waste in India

**DOI:** 10.1038/s41598-018-21326-9

**Published:** 2018-02-13

**Authors:** Chander Kumar Singh, Anand Kumar, Soumendu Shekhar Roy

**Affiliations:** Department of Energy and Environment, TERI School of Advanced Studies, New Delhi, India

## Abstract

Increased emissions of greenhouse gases have altered the global ambient temperature and adversely affected global climatic conditions. The municipal solid waste (MSW) generated by households is considered the third largest anthropogenic source of methane (CH_4_) emissions, constituting 11% of all global CH_4_ emissions. The current study derived total MSW CH_4_ emission estimates using the IPCC default method (DM), modified triangular method (MTM) and first order decay method (FOD). The estimated CH_4_ emission was higher for the DM than the other methods, and was comparable to estimates from other studies. This study observed that the net annual emission of CH_4_ from landfills in India increased from 404 Gg in 1999–2000 to 990 Gg and 1084 Gg in 2011 and 2015, respectively. We also found that CH_4_ emissions were highly correlated (R^2^ = 0.8) with the gross state domestic product (GSDP) of states and the gross domestic product (GDP) of the country, which is an indicator of human well-being. The MSW management policy of India needs to be reviewed in a current policy context, as the management and efficient utilization of MSW technologies might help increase the use of CH_4_ as an energy source and thereby improve its sustainable and cost-effective management.

## Introduction

The increase in greenhouse gas (GHG) emissions has altered the global temperature pattern and created a threat against human health and the environment^1–6^. Methane emitted from landfills is one of the most important contributors to GHGs. Methane (CH_4_) is regarded as one of the most important GHG because of its global warming potential, which is 28 times higher than that of CO_2_^7^ over 100 years. Over the last few centuries, the concentration of CH_4_ in the atmosphere has increased rapidly. In a span of 260 years, from 1750 to 2010, the concentration of GHG CH_4_ in the environment has increased from 700 ppb to 1808 ppb^7^. The rate of increase observed during the last few decades is 1–2% per year^8,9^. Along with natural sources, i.e., wetlands, termites, and oceans, which account for 36% of the total CH_4_, anthropogenic activities, including the production and burning of fossil fuels, livestock farming, landfills and agriculture, contribute 64% of the total amount of global CH_4_ emissions^10–15^. Rapid population growth and development activities have increased the potential CH_4_ emission level, and it has been estimated that the concentration of GHG CH_4_ is expected to increase from 6.88 Gt CO_2_-eq, the value recorded in 2010, to 8.59 Gt CO_2_-eq by 2020^16^. The decomposition of municipal solid waste (MSW), i.e., wastes generated from households and residential settings^17^, is considered the third major anthropogenic source of CH_4_, and it contributes approximately 11% of the total anthropogenic CH_4_ emissions^18^. Once MSW is disposed into landfills, it undergoes aerobic decomposition, which produces a very small amount of CH_4_. Afterwards, the anaerobic condition prevails, and due to methanogen activities, the MSW emits CH_4_ for years, even if the landfill is closed^15^. The technologies used to generate energy from MSW that are prevalent in India, are incineration and biochemical conversion^19–23^. In India, only 70–75% of the MSW gets collected, and only 20–25% of it is treated. The technologies involved in MSW management include composting/vermicomposting, recycling and landfilling. So far, only 553 compost and vermicompost plants, 56 bio-methanation plants, 22 refuse derived fuel (RDF) plants and 13 waste-to-energy conversion plants are installed in the country (CPCB 2013–14)^24^. Many of these schemes have experienced failure due to several issues related to the segregation of waste, low calorific values of the waste, and challenges in the operation and maintenance of the plants (CPCB 2013–14).

Asian countries are the largest producers of MSW due to their high population densities. The generation of MSW in Asia is predicted to increase up to 1.8 million tons/day by 2025 from the current value of 1 million tons/day^25^. India, with its huge population and GDP growth rate of 6.7%, is witnessing rapid urbanization and lifestyle changes. The per capita generation rate of MSW in India ranges from 0.2 to 0.5 kg/day^26^. Presently, approximately 90 million tons of solid waste is generated by the country, which is eight times higher than the amount of solid waste that was generated in 1947^27^. Metropolitan cities have the largest contribution to the generation of waste compared to smaller and less developed cities. At present, India produces 16 Mg CO_2_ equivalent of CH_4_ per year, and the annual value is expected to reach 20 Mg CO_2_ equivalent by 2020. It is also estimated that CH_4_ contributes 29% of the total GHG emissions from the country, which is higher than the global average of 15%^27,28^. Studies suggest that the MSW generated in India mostly consists of a large fraction of organic wastes (40–60%), 3–6% paper waste, and 30–40% ash and fine earth material waste, with smaller fractions of plastics, glass and metal wastes (all <1%). The moisture content of the wastes is 47%, with an average calorific value of 6.8–9.8 MJ/kg and a C/N ratio of 800–1000 Kcal/kg^21,23,26^.

In India, due to inadequate data availability, a large amount of uncertainty related to MSW management and emissions has been observed, which makes it difficult to estimate the accurate value for the landfill GHG emission potential. The International Panel on Climate Change^29^ has established a method for the estimation of GHGs emitted from landfills that has been widely used by researchers^9,27,30,31^.

Studies have been conducted by using different methods, i.e., the stoichiometric method^32^, default method (DM)^9,33^, first order decay method (FOD), modified triangular method (MTM)^33,34^, *in situ* closed chamber methods^35,36^ and the landfill gas emission method (Land GEM)^27^. India is the largest producer of MSW, and landfills are third largest contributors to the total CH_4_ emission value of the country. To reduce the total CH_4_ emission value, a strong policy narrative is required in India. The CH_4_ emitted from landfills can be utilized as a potential source of renewable energy. With the above background, this study aimed to estimate the potential emissions of CH_4_ from landfills at the national scale, which would support sustainable management practices in a quickly evolving economy.

## Results

### Temporal CH_4_ emission estimates with the DM, MTM and FOD

**Figure 1:**
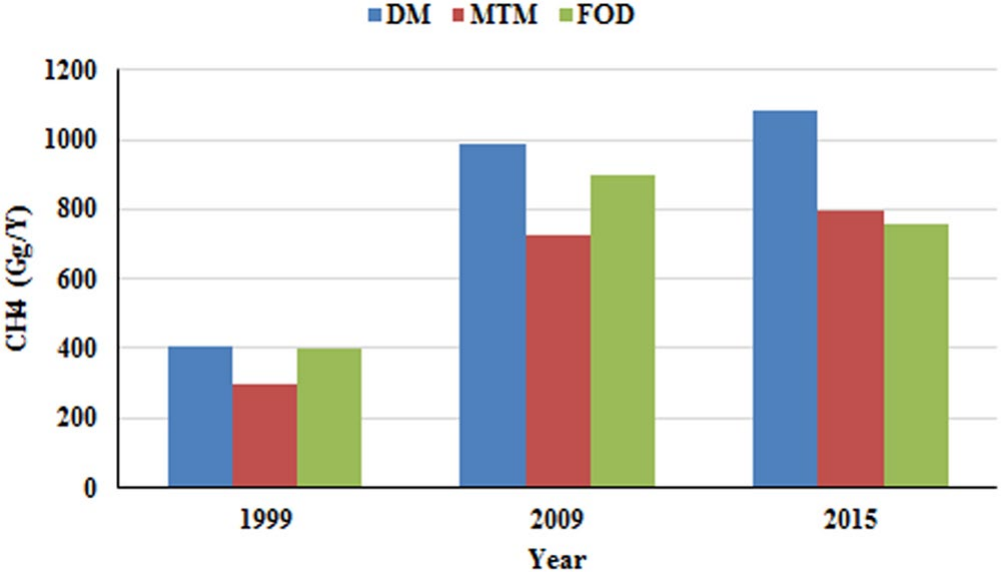
Estimated CH_4_ emissions from MSW in India from 1999–2015 using the DM, MTM and FOD.

The CH_4_ emission values calculated using the DM, MTM and FOD are given in Fig. 1. Using the DM and TM, Kumar *et al*.^9^ estimated the net annual methane generated in India to be 502.46 Gg and 400.66 Gg, respectively, in the year 1999^9^. However, this estimation was done for Class I and Class II cities. The current study estimates the CH_4_ emission for the entire country, except for the states of Nagaland, Sikkim, Uttaranchal, Arunachal Pradesh, the Andaman and Nicobar Islands, Chhattisgarh, Goa, Daman and Diu, Lakshadweep and Gujarat for the year 1999. Out of these, three states were created in November 2000. Gujarat, a major contributor of CH_4_ emissions, was not included in the study due to an absence of data for the year 1999. Our estimate with the DM, which is 404.86 Gg, used a fraction of degradable organic carbon (DOC) of 0.114 instead of 0.16, the value used by Kumar *et al*.^9^, based on the fractions depicted in the CPCB, 2013. However, when the DOC value of Kumar *et al*.^9^ was used, the DM produced a CH_4_ emission estimate of 625.05 Gg/Y. This study had different values compared to those of Kumar *et al*.^9^, because it considered the total MSW contribution at a national scale instead of only considering major cities. Similarly, the MTM of this study estimated a lower value, 297.52 Gg/Y, than the triangular method used by Kumar *et al*.^9^. The FOD yielded a CH_4_ emission value of 402.39 Gg/Y for 1999. The total CH_4_ emission value of three landfill sites in Delhi was calculated by Chakraborty *et al*.^34^, for the year 2009 using the DM (45.7 Gg), MTM (41.4 Gg) and FOD (31.1 Gg)^34^. However, these estimates were for the MSW received by three landfill sites, not the entire MSW generated in Delhi. The values obtained in our study were 57.35 Gg, 42.14 Gg and 52.15 Gg for the same year using the DM, MTM and FOD methods, respectively, for the entire amount of MSW generated in Delhi rather than the MSW of the three landfill sites considered in Chakraborty *et al*.^34^. Thus, we found that the DM of the IPCC and the FOD estimates of the CH_4_ emissions agree with estimates from earlier studies.

The amount of CH_4_ emissions increased approximately 2.5 times in a span of 10 years (1999–2009), reaching a total emission value of 1084.03 Gg/Y by 2015. An increase of 245% is observed from the year 1999 to 2011, while a total increase of 109% was found from 2011 to 2015 (Fig. 1).

### Trends in methane emissions from landfills and populations

In the past few decades, India has witnessed rapid population growth in urban areas; thus, the overall population has grown (Table 1).

**Table 1:** Population growth of India (decadal).

S. No	Census Year	Population (in Millions)	Decadal Growth Rate (%)
1	1911	252.09	5.75
2	1921	251.32	−0.31
3	1931	278.98	11.00
4	1941	318.66	14.22
5	1951	361.09	13.31
6	1961	439.23	21.51
7	1971	548.16	24.80
8	1981	683.33	24.66
9	1991	846.30	23.85
10	2001	1028.61	21.34
11	2011	1210.19	17.64

Migration from rural areas to the urban areas has increased the generation of MSW. According to the census of India, during the period from 2001 to 2011, the population of the country increased by 181 million, which is 17.6% of the total population, and within the 2001–2011 period, the urban population increased by 31.8%^37^. This increase in the urban population has resulted in a huge generation of MSW and has also changed the characteristics of the wastes due to changes in the economic status and living standards of the population.

### Spatial distribution of CH_4_ and gross state domestic product

**Figure 2:**
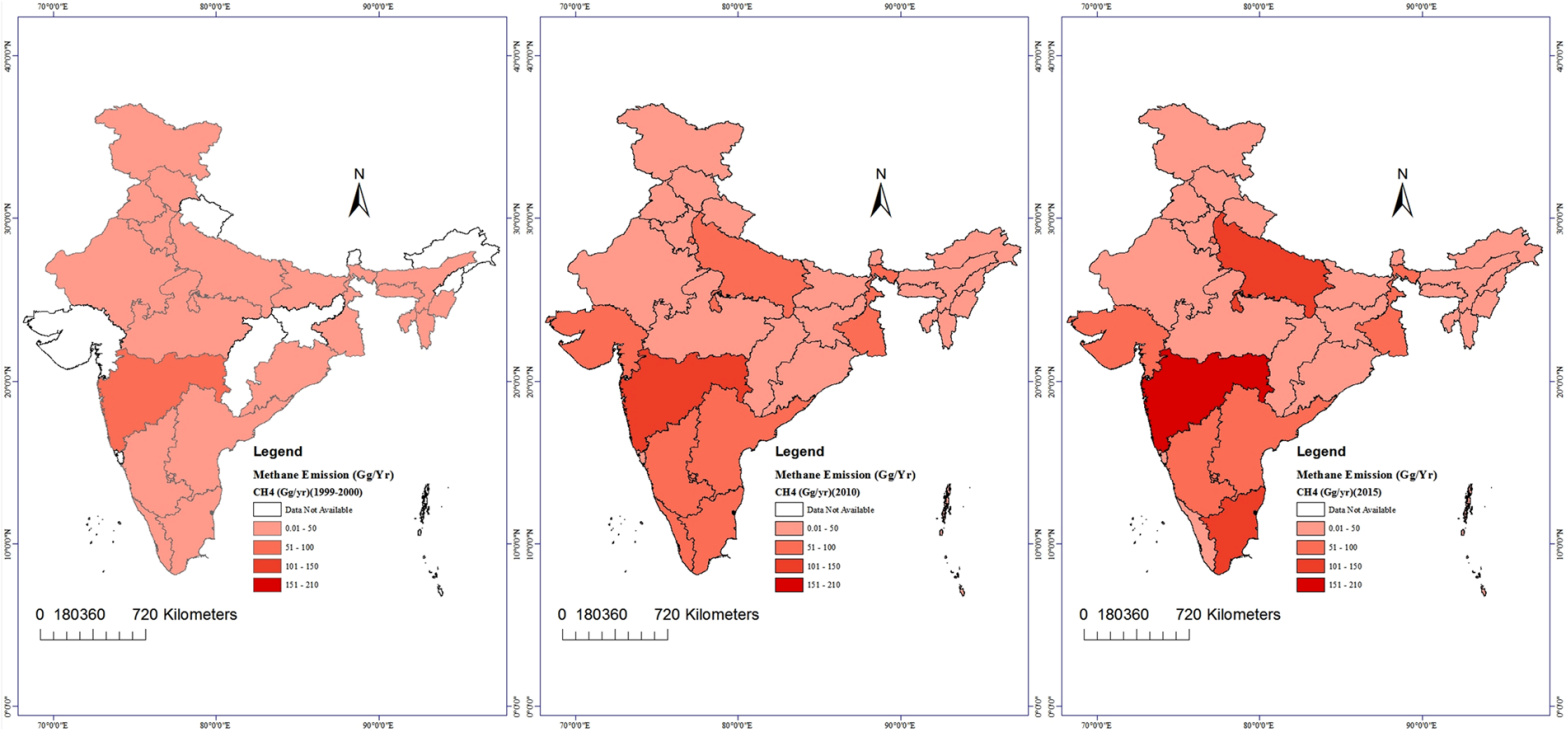
Spatial distribution (state-wise) of the CH_4_ in the periods 1999–2000, 2009–10 and 2014–15 using the spatial analyst extension of Arc GIS 10.1.

**Figure 3:**
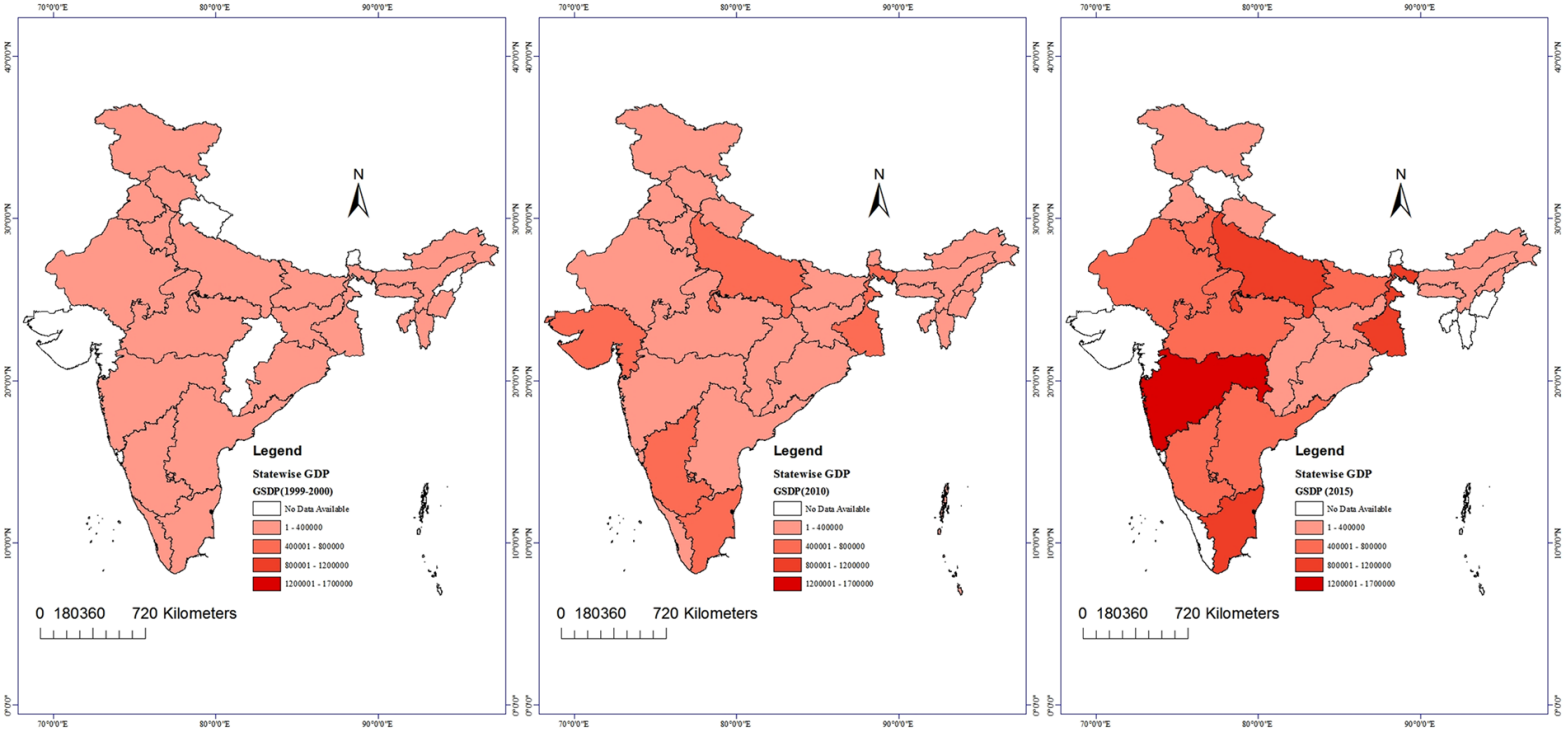
Spatial distribution (state-wise) of the GSDP in the periods 1999–2000, 2009–10 and 2014–15 using the spatial analyst extension of Arc GIS 10.1.

In the period from 1999 to 2000, a total of 404 Gg CH_4_ was released into the atmosphere from the MSW in landfills. The maximum CH_4_ emission was observed for Maharashtra (70.6 Gg), while the minimum emission was observed for Tripura (0.2 Gg). From 2009 to 2010, 990 Gg of CH_4_ was released, and Maharashtra was the leading contributor, with 149 Gg of emissions, followed by West Bengal (97.5 Gg), Tamil Nadu (97.1 Gg) and Uttar Pradesh (89.9 Gg) (Figs 2, 3).

In 2015, a total of 1084 Gg CH_4_ was released, with the maximum contribution, 208 Gg, emitted from Maharashtra, followed by Uttar Pradesh (148 Gg) and Tamil Nadu (112 Gg). The results of this study are similar to those of Kumar *et al*.^9^, who reported net annual CH_4_ values of 400 Gg and 500 Gg in 1999 using the direct and first order decay (FOD) method, respectively (Table 2).

**Table 2:** Maximum CH4 (Gg/yr) producer states and their GSDP (in crore).

Year 1999–2000			Year 2009–10			Year 2014–15		
States	GSDP	CH_4_ Emission (Gg)	States	GSDP	CH_4_ Emission (Gg)	States	GSDP	CH_4_ Emission (Gg)
Maharashtra	247830	70.67	Maharashtra	1170121	149.16	Maharashtra	1686695	208.32
Uttar Pradesh	175159	46.29	West Bengal	528316	97.53	Uttar Pradesh	976297	148.97
Tamil Nadu	134185	41.97	Tamil Nadu	667202	97.12	Tamil Nadu	976703	112.87
West Bengal	135376	35.89	Uttar Pradesh	685496	89.98	Andhra Pradesh	520030	89.32
Andhra Pradesh	128797	33.99	Andhra Pradesh	362245	89.32	Gujarat	NA	71.67

**Figure 4:**
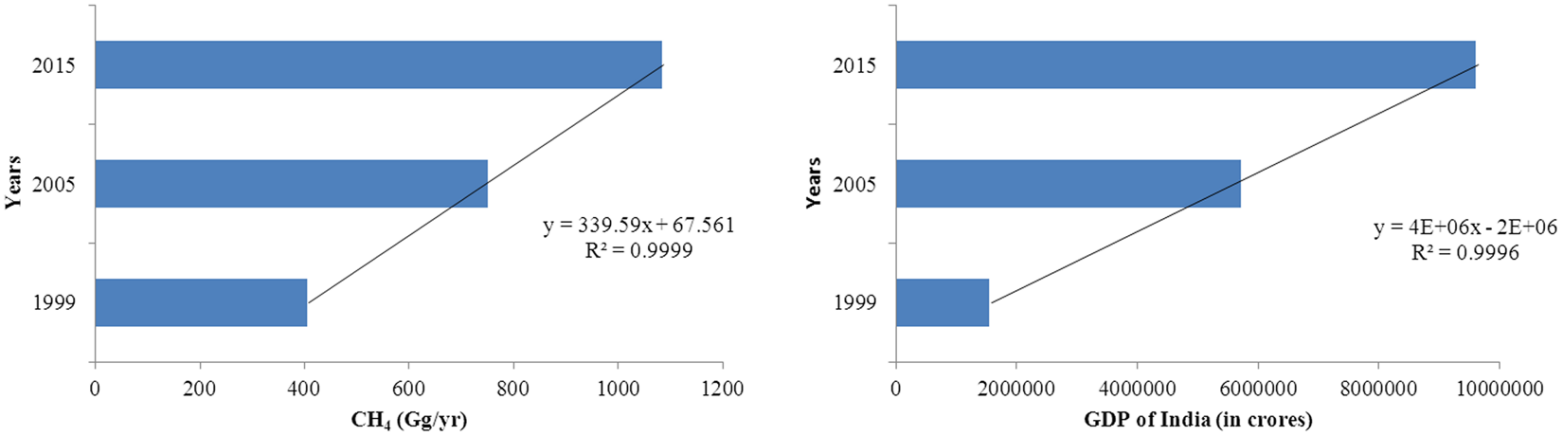
CH_4_ generation from landfills (Gg/yr) and the GDP (crores) of India from 1999–2015.

The generation of MSW is often linked with per-capita income and development. Our finding shows a very high correlation between the MSW methane emissions and the GSDP of the states, which is considered an indicator of social well-being (Fig. 4).

**Figure 5:**
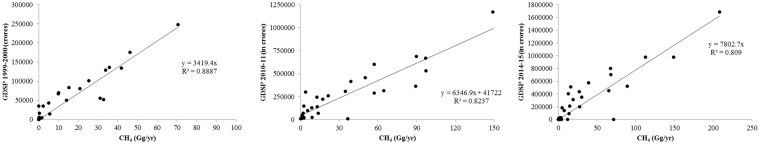
Correlation between the GSDP and CH_4_ emissions.

Based on the available data for the periods 1999–2000, 2009–2010 and 2014–15, along with the GSDP data for the same years, it has been observed that the generation of CH_4_ is very much related to the population and GSDP of the states (Fig. 5). The positive association, based on *R*^2^, between the GSDP and the CH_4_ emissions from landfills was 0.88, 0.68 and 0.80 for 1999–2000, 2009–10 and 2014–15, respectively. A high GSDP indicates high levels of daily human activities and consequently a high amount of MSW generation, leading to CH_4_ emissions.

## Discussion

### Methane Emission, Population Growth and Economic Development

We found that the CH_4_ emissions were highly correlated with economic development and population growth. Economic development drives the population to move to cities where basic infrastructure and amenities are available. This diversion of the population subsequently leads to changes in the overall lifestyle and living standards and thereby increases the per capita generation of MSW. We found that the higher the population density, the more MSW was generated. Similarly, for the regions, higher GDPs indicated higher human activity levels and consequently higher MSW generation rates. The generation of MSW is often linked with per-capita income and development. The generation of MSW is considered high in developed countries compared to developing countries^30^. Our findings show a high correlation between the MSW methane emission level and the GSDP, which is an indicator of social well-being, of the states. Along with their development level, the lifestyles of the communities also change accordingly, which further enhances the generation of MSW.

### CH_4_ from landfills: A potential source of energy

India, one of the fastest growing economies, is still improving its existing technological capability to generate power sources and energy from MSW. Most of the gases emitted from landfills have the potential to be utilized as biogas, which is a source of clean/green energy. Landfills directly emit GHGs into the environment, which affects the global temperature and puts the environment at risk. The open dumping of wastes leads to significant reductions in nutrients, i.e., nitrate, potassium and phosphorus; reduces the ability to recycle and reuse waste; and increases the risk of groundwater pollution by leaching. Controlled or engineered landfills with a proper covering and collection system can enable the sustainable disposal of solid wastes and their use as an energy source. According to one estimate, only 20–25% of landfill gases can be recovered, while the rest of the gases escape into the atmosphere. If properly designed landfills are established^38^, 30–60% of the gases can be utilized from these controlled landfills^39^. These recovered landfill gases can be used as a direct source of energy and for power generation or can be upgraded into vehicle fuel^27^.

### Status of and challenges for MSW management in India

The emission of CH_4_ is directly related to the amount of waste generated from the households or the society in a region. In India, there is a huge amount of climatic, cultural and demographic variability based on the different food habits of its communities. According to the 2011 census, the urban population in India increased from 285 million in 2001 to 377 million in 2011, and approximately 70% of this population resides in 366 cities of the country. In the present study, we observed that over the last fifteen years, the total MSW has increased by three times compared to the amount that was generated in 1999–2000, i.e., from 52125 MT/day in 1999–2000 to 127485 MT/day in 2010 and 139566 MT/day in 2015. Landfills are one of the most cost effective, simplest, and oldest techniques for MSW management, but they require land area near cities but away from habitation clusters and other places of socio-economic or environmental importance (MoEF, 2000). Most of the existing landfills in megacities such as Delhi are oversaturated and are serving beyond their maximum carrying capacity. When a landfill operating above its capacity in Delhi collapsed on 1st September 2017, a 50 m high mound of waste killed several people^40^. According to one estimate, the total urban waste generated in India will increase to 107 thousand tons/year in 2031 and will reach up to 160.9 thousand tons/year in 2041. The proper disposal of this waste will require an area of 1400 sq.km, i.e., an area equivalent to the area of Hyderabad, Mumbai and Chennai^21^.

MSW contains large amounts of biodegradable wastes and wastes with high calorific values that can be utilized for vermicomposting/compost preparation and energy generation, respectively. The Indian government revised the 2001 law related to MSW after 15 years and produced a new MSW law in 2016 under the Environmental (Protection) Act of 1986 for the proper management and utilization of MSW. This law is intended to make the community aware of ways to reduce waste generation and to involve locals in the proper management of waste and innovations of technology for recycling and energy recovery purposes. The huge amount of waste generated in the country has a potential use as renewable energy, as landfill gases have high a calorific value, and can consequently minimize the impacts of greenhouse gas emissions on the climate.

## Conclusion

In the last few decades, the overall growth of the population, urbanization level and economy of India has increased the amount of municipal solid waste generated in the country. The disposal of these wastes into landfills results in the emission of gases with a high global warming potential such as CH_4_, CO_2_, and NO_x_. The results of this study indicate that a gradual increase in the CH_4_ emissions has occurred over the last 15 years (1999–2015). This study also establishes a relationship between the emission of CH_4_ and the well-being of the associated community, which is highly linked to the gross state domestic product (GSDP) of the states. Landfill gases have the potential to be utilized as sources of green energy, and if properly planned and engineered landfills are constructed at suitable sites, these gases can be recovered and utilized as a sustainable source of renewable energy.

## Methods

### Data acquisition and determination of waste characteristics

This study was conducted to determine the generation and composition of waste in various parts of the country. It has been observed that the composition of waste depends on various factors, including the food habits and socio-cultural practices of a community, the climate and the community income^41,42^. The source composition of MSW in most Indian cities consists of a fraction of organic matter varying from 40 to 60%, followed by fractions of earth and ash materials (30–40%), paper (3–6%) and plastic, glass and metals (1% each)^27^. Kumar *et al*.^9^, reported that the composition of municipal waste was 30–70% rapidly biodegradable organic matter, 0.6–31% paper and cardboard, and 1–16% plastic materials^9^. In this study, we assume that 70% of the MSW reaches a landfill. The collection efficiency in India is poor due to a number of reasons (e.g., poorly designed bins, open dumps, collection vehicles, and the frequency of waste collection). The average collection efficiency for MSW in Indian cities and states is approximately 70%^23,43,44^. The MSW generation data for the periods 1999–2000 and 2009–2012 were collected from the Central Pollution Control Board (CPCB 2013, 2016), while the GDP data for the same years was taken from the Central Statistics Office, Government of India. The population data of the country was taken from the Census of India^37^.

### Calculation of CH4 emissions from landfills

Among the available methods for the estimation of CH_4_ emissions from landfills, the simplest one was provided by Bingemer and Crutzen (1987)^45^ and revised by the IPCC in 1996. It is a mass balance approach that provides the actual emissions from the landfill, and it is widely used when detailed data are not available. The CH_4_ emissions from MSW were calculated in accordance with Kumar *et al*.^9^:

1 $$C{H}_{4}\,(Gg/yr)=(MS{W}_{T}\times MS{W}_{F})\times MCF\times DOC\times DO{C}_{F}\times F\times (\frac{16}{12}-R)\times (1-OX)$$

where 1 Gg/yr = 1000 tons/yr.

In equation 1, MSW_T_ = total amount of solid waste generated (Gg/yr),

MSW_F_ = the fraction of MSW that reaches the landfill sites (considered 70% for the current study, as the rest of the MSW was considered lost by recycling, reuse or other processes),

MCF = Methane correction factor, and

DOC = Degradable organic carbon, which can be calculated using the following equation:

2 $${\rm{DOC}}=0.4{\rm{A}}+0.17{\rm{B}}+0.15{\rm{C}}+0.3{\rm{D}}$$

where A = paper, cardboard and rags, B = leaves, straw and others, C = fruit and vegetable wastes and D = wood,

DOC_F_ = the value of the dissimilated fill gas, which was considered 0.77 (at a temperature of 35 °C^9,10^),

F = fraction of methane gas, which was considered 0.5,

R = recovered methane gas, which was considered 0 for this study, as recovery techniques for landfill gases have not been adopted in most of India^9^, and

OX = oxidation factor, which was also considered 0 (as the default value) and accounted for the methane gas that was oxidized in the upper layer of the waste in the presence of oxygen.

The IPCC has identified two types of uncertainties in the model, but the model generates reasonable estimates for regions where the data on waste generation are limited.

The uncertainty in the model is as follows: First, the methodology assumes that a constant amount of waste is added each year and that the CH_4_ generation is the same in each year. Thus, temporal increments at the landfills are not considered, which may cause the methane emission value to be overestimated. The amount of waste disposed is a highly sensitive parameter in the default methodology.

Second, uncertainties in the estimates of the MSW_T_ and MSW_F_ may cause greater uncertainties in the total methane emission estimate. The model is also sensitive to the waste composition; the DOC content is sensitive to small variations in the assumed values, which can change the overall methane emission estimate.

**Figure 6:**
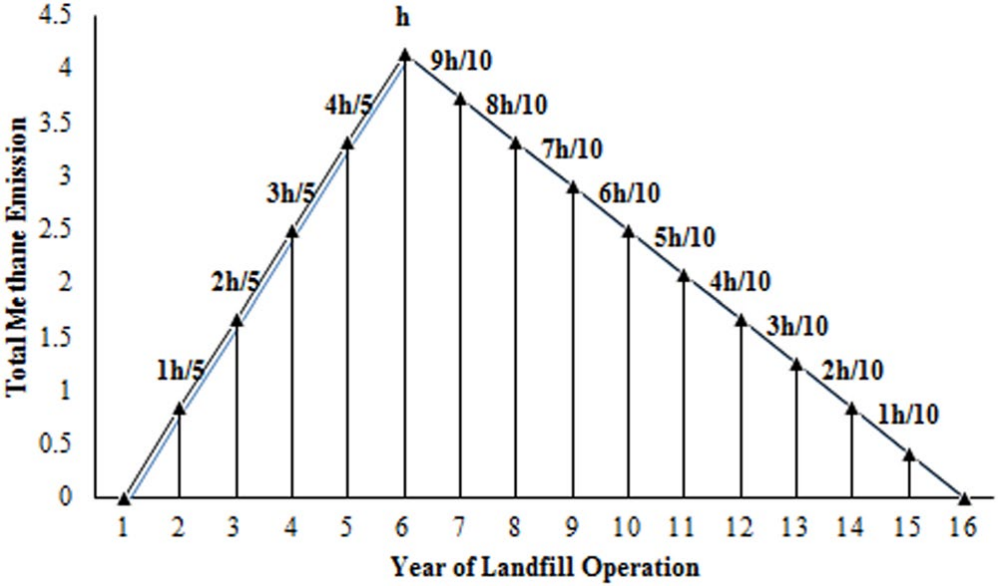
Triangular form of gas generation.

Modified Triangular Method: The MTM is best suited for regions where the waste characterization data are not appropriate/adequate. This method considers the waste degradations to occur in two phases: first, the waste degradation starts one year after the deposition of the waste, and the rate of gas generation reaches a peak within the first 6 years. In the second phase, the gas generation decreases linearly to zero by the 16^th^ year (Fig. 6). The amount of gas released is based on the FOD in triangular form, as shown in Fig. 6, for the emission estimate for Delhi. The total gas generation (G) during the period *t* + 1 to *t* + 16, where t is the year of the waste deposition, is given by:

${\rm{G}}=1.87{{\rm{A}}}_{{\rm{t}}}{{\rm{C}}}_{0}$ where A_t_ is the amount of the waste deposited in year *t*.

The FOD estimates the temporal change in methane emissions. The underlying assumption in this method is that the DOC decays steadily with CH_4_ formation. Limited data on the MSW can be analyzed by the FOD. The methane emissions can be calculated as follows:

3 $${E}_{CH4}=\sum _{T}(({e}^{-(T-1)k}-{e}^{Tk})\,\ast \,MSW\,\ast \,MCF\,\ast \,DOC\,\ast \,DO{C}_{F}\,\ast \,F\,\ast \,\frac{16}{12}-R)\,\ast \,(1-OX)$$

4 $$K=\frac{ln2}{{t}_{\frac{1}{2}}}$$

where E_CH4_ represents the methane emissions from an MSW landfill,

T is the inventory year for which the emissions are calculated,

k is the reaction rate constant,

t_1/2_ is the half-life of methane,

MSW is the total MSW disposed in the landfill,

MCF is the methane correction factor,

DOC is the fraction of degradable organic carbon,

DOC_F_ is the fraction of total DOC that degrades,

F is the fraction of methane in the landfill gas,

16/12 is the conversion ratio (CH_4_/C),

R is the methane recovered value, and

OX is the oxidation factor. The values of the parameters used here were derived from the IPCC^8^.
